# Comparative Effectiveness of an Artificial Air Pocket Device to Delay Asphyxiation in Supine Individuals Critically Buried in Avalanche Debris

**DOI:** 10.1001/jamanetworkopen.2023.13376

**Published:** 2023-05-15

**Authors:** Giacomo Strapazzon, Simon Rauch, Sandro Malacrida, Tomas Dal Cappello, Enrica Governo, Bruna Catuzzo, Simona Mrakic-Sposta, Margherita Urgesi, Marika Falla, Gianluca Cavoretto, Enrico Visetti, Guido Giardini, Hermann Brugger, Federico Prato

**Affiliations:** 1 Institute of Mountain Emergency Medicine, Eurac Research, Bolzano, Italy; 2 Corpo Nazionale Soccorso Alpino e Speleologico, Milano, Italy; 3 Department of Anesthesia and Intensive Care Medicine, F. Tappeiner Hospital, Merano, Italy; 4 Mountain Medicine Center, Azienda Sanitaria Valle d’Aosta, Aosta, Italy; 5 Institute of Clinical Physiology, National Research Council, Milano, Italy; 6 Center for Mind/Brain Sciences, University of Trento, Rovereto, Italy; 7 Department of Anesthesia and Intensive Care, Gruppo Policlinico di Monza, Monza, Italy; 8 Department of Emergency Medicine, Azienda Ospedaliero-Universitaria Maggiore della Carità, Novara, Italy

## Abstract

**Importance:**

Approximately 70% of individuals critically buried in avalanche debris die within 35 minutes as a result of asphyxial cardiac arrest. An artificial air-pocket device (AAPD) that separates inhaled air from exhaled air may delay the onset of severe hypoxemia and eventual asphyxia during snow burial.

**Objective:**

To investigate the efficacy of a new AAPD during snow burial in a supine position.

**Design, Setting, and Participants:**

This comparative effectiveness trial was performed in winter 2016 with data analysis in November 2016 and November 2022. Each trial used a simulated critical avalanche burial scenario, in which a trough was dug in a snow pile and an additional air pocket of 0.5 L volume was punched into the lateral wall for each control trial. All participants were buried in a supine position. Trials could be voluntarily terminated at any time, with a maximum length of 60 minutes; trials were automatically terminated if the participant’s peripheral oxygen saturation (Spo_2_) dropped to less than 84%.

**Exposures:**

Each participant conducted 2 trials, one in which they breathed into the AAPD (intervention trial) and the other in which they breathed into the prepared air pocket (control trial).

**Main Outcomes and Measures:**

Measurements included Spo_2_, cerebral oxygenation, ventilatory parameters, respiratory gas concentrations, and visual-analogue scales. Kaplan-Meier survival curves and rank test for matched survival data were used to analyze the total burial time in each trial.

**Results:**

A total of 13 volunteers (9 men; mean [SD] age, 33 [8] years) were exposed to the intervention and control trials. Intervention trials were terminated less often (2 of 13 trials) as a result of hypoxemia than control trials (11 of 12 trials). Similarly, survival curves showed a longer duration of burial in the intervention compared with the control trials for the time to reach an Spo_2_ less than 84% (rank test for matched survival data: *P* = .003). The intervention trials, compared with the control trials, also had slower rates of decrease in fraction of inspired oxygen (mean [SD] rate, −0.8 [0.4] %/min vs −2.2 [1.2] %/min) and of increase in fraction of inspired carbon dioxide (mean [SD] rate, 0.5 [0.3] %/min vs 1.4 [0.6] %/min) and expired ventilation per minute (mean [SD] rate, 0.5 [1.0] L/min^2^ vs 3.9 [2.6] L/min^2^).

**Conclusions and Relevance:**

This comparative effectiveness trial found that the new AAPD was associated with delaying the development of hypoxemia and hypercapnia in supine participants in a critical burial scenario. Use of the AAPD may allow a longer burial time before asphyxial cardiac arrest, which might allow longer times for successful rescue by companions or by prehospital emergency medical services.

## Key Points

**Question:**

Can an artificial air pocket device (AAPD) prevent severe hypoxemia and eventual asphyxia in individuals in a supine position during a simulated critical avalanche burial?

**Findings:**

In this comparative effectiveness trial including a cohort of 13 healthy participants, the intervention trials (breathing into AAPD) were terminated consistently less often as a result of hypoxemia than the control trials. Survival curves showed a significantly longer duration of burial in the intervention trials compared with the control trials.

**Meaning:**

These findings suggest that AAPD use may allow a longer burial time before asphyxial cardiac arrest in individuals critically buried in snow, which in turn might allow longer times for successful rescue attempts by companions or by prehospital emergency medical services.

## Teaser

This comparative effectiveness study assesses an artificial air-pocket device vs a placebo control to delay onset of hypoxia during snow burial in a supine position.

## Introduction

The increasing popularity of outdoor winter activities has led to increased numbers of avalanche injuries among winter recreationists. Survival analysis has shown that 70% of individuals critically buried in avalanches die within 35 minutes as a result of asphyxial cardiac arrest.^1^ The onset of asphyxia is related to airway patency and the presence of an air pocket (defined as an air space in front of the mouth and nose), its volume, and snow characteristics.^2,3,4^ The accumulation of carbon dioxide (co_2_), owing to the rebreathing of exhaled air, accelerates the development of hypoxia.^5^ Devices have been developed to increase the survival chances of people buried in avalanches by preventing hypercapnia and therefore slowing hypoxemia. Artificial air pocket devices (AAPDs) are designed to separate exhaled air from inhaled air. In a 2000 experimental snow burial study,^5^ 8 participants sustained adequate oxygenation for up to 60 minutes in the intervention trials (breathing into an AAPD) while buried in a seated position, whereas in the control trials without the AAPD, participants terminated much earlier (after approximately 10 minutes). In another similar experimental study, 8 participants sustained adequate oxygenation for up to 90 minutes when expired co_2_ was removed from the surrounding snowpack.^6^ However, individuals critically buried in avalanches are most often found in a prone position (45%), or supine position (24%).^7^ The supine position may even promote asphyxia by either directly compromising chest wall expansion as a result of the additional weight of the snow on the thorax or indirectly limiting caudal diaphragm movement.^1^ To our knowledge, no data on the efficacy of an AAPD or ventilatory parameters in supine participants have been reported.

As a result, the primary aim of this study was to investigate the efficacy of a new AAPD during snow burial in a supine position. The secondary aim was to describe the physiological and ventilatory parameters under simulated avalanche snow debris. In a comparative effectiveness trial, participants either breathed into the AAPD in the intervention group or into an air pocket in the control group, within a crossover design.

## Methods

This comparative effectiveness trial was approved by the institutional review board of Azienda USL Valle d’Aosta. We conducted the study in adherence to the Declaration of Helsinki. All participants were informed about the possible risks of being fully buried and gave written informed consent prior to enrollment. This study is reported following the Consolidated Standards of Reporting Trials (CONSORT) reporting guideline.

### Study Population

The recruited participants were healthy, unpaid, mountain enthusiasts, mountain rescuers, and health care practitioners. Participants of both sexes, with age at least 18 years and classified according to the American Society of Anesthesiologists (ASA)^8^ as class I were considered eligible. Exclusion criteria were age younger than 18 years, ASA class greater than I, and any acute disease.

### Study Protocol

Each participant performed 2 trials (intervention and control) in a crossover design with a minimum of 1 overnight washout between the 2 trials. A randomization list was created, but we were not able to fully respect the randomization list due to unpredictable weather that changed environmental conditions. This meant that in most trials, the intervention trials preceded the control trials.

**Figure 1.**
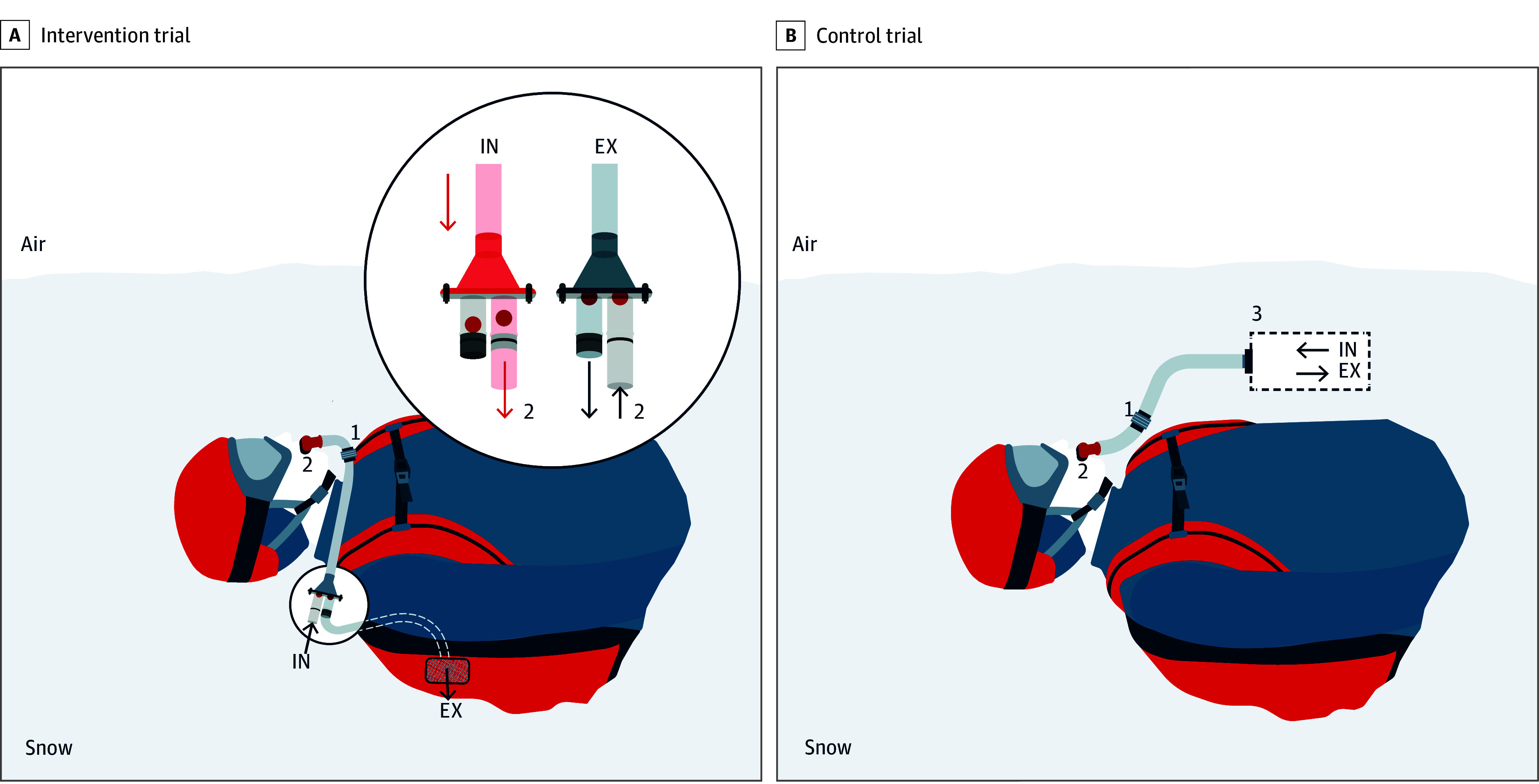
Experimental Setup A, On inhalation (IN), air from the avalanche debris enters the inhalation/exhalation separator through the first of two 1-way valves (red dots). Air then flows through the respiratory tubing, passes the in-line ergospirometer (1) into the airway (2). On exhalation (EX), air goes away from the airway, again passes the ergospirometer (1), and then through the IN/EX separator exits through the exhalation tube. B, On IN, air from the air pocket flows through the respiratory tubing, passes the in-line ergospirometer (1) into the airway (2). On EX air again passes the ergospirometer (1) and is directed through the exhalation tube back into the air pocket (3). Air, white; snow, gray. Illustration by Eurac Research/Silke De Vivo. All rights reserved, used with permission.

In the intervention trial, each participant breathed into a new AAPD (Airsafe, Ferrino). The AAPD separates inhaled air from exhaled air using two 1-way antifreeze-ball valves, directing the exhaled air behind the user into the snowpack (Figure 1). Whereas, in the placebo control trial, each participant breathed into the AAPD mouthpiece, but instead of being connected to the AAPD, it was connected to the snow air pocket (volume, 0.5 L). Each participant received a standardized training on the use of the AAPD prior to the trial start and remained blinded to the trial condition.

Participants were buried in a supine position, with both their head and chest under simulated avalanche debris (critical burial) for a maximum of 60 minutes. Trials were either voluntarily terminated by participant request (pulling a rope attached to the hand), or when peripheral oxygen saturation (Spo_2_) dropped to less than 84%. Alternatively, if required, the trial could be terminated by the principal investigator for other medical (end-tidal co_2_ [etco_2_] >70 mm Hg, tachycardia >150 beats per minute, or arrhythmias) or technical (eg, failure of the monitoring system) reasons.

**Figure 2.**
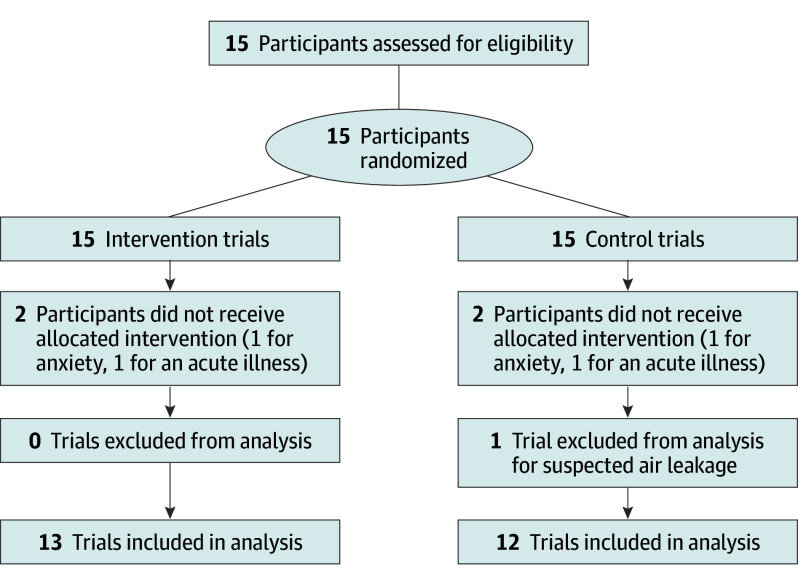
Participant Flowchart

The participant flowchart is illustrated in Figure 2. Measurements were taken indoor for baseline and in the field for trial measurements. Before each trial, participants rested for approximately 20 to 30 minutes in a warm environment to minimize anxiety, stress, and cold exposure and to increase familiarization with the device.

### Setting and Instrumentation

All trials were performed at Plan Maison, Valtournenche, Italy (elevation, 2561 m) in winter 2016. The experimental setup simulated a critical avalanche burial. Each trial day, a snow pile mimicking an avalanche deposit was prepared with fresh snow. After snow sintering, a trough was dug in the snow pile. A cylindrical air pocket of 0.5 L volume was punched in the lateral wall of the snow trough for each control trial. The pocket was closed with a well-fitted, custom-made plastic cover complete with a connection to fix the tubing system. The volume of the air pocket was chosen based on previous literature.^2,4,5^

Participants breathed into the anatomical mouthpiece of the AAPD connected to a modified tubing system (Figure 1), which allowed continuous monitoring of ventilation and respiratory gases by a portable telemetric gas analysis system (K4b2, Cosmed). The gas analysis system was placed in line with the tubing between the mouthpiece and the housing containing two 1-way valves in the intervention trial or between the mouthpiece and the air pocket in the control trial. The dead space of the tubes was the same in both the intervention and control trials.

Each participant wore their own thin base layer and mid-layer in combination with a thick, hooded snowsuit shell, which was provided along with a standardized set including a face mask, goggles, and gloves. All participants in both trials were fitted with the same backpack with an integrated tubing system. An emergency oxygen line was placed near the participants airway to ensure their safety and comfort if they chose to be extricated early.

Each participant was placed in supine position in the snow trough. The nose was occluded with a nose clip to ensure mouth breathing only. At time zero, the main investigator (G.S.) put the mouthpiece into the participant’s mouth and the participant started to breathe directly into the tubing system. Simultaneously, 2 study investigators shoveled the prepared snow onto the participants from above. The trough was filled with snow within approximately 30 seconds. Burial depth was reproduced for each trial and amounted to at least 50 cm at the participant’s head and chest.

### Measurements

Physiological parameters that were continuously measured noninvasively included heart rate (HR), as derived from 3-lead electrocardiogram (Lifepack15, Physio-Control); Spo_2_, both from a forehead (Lifepack15) and finger sensor (Radical-7, Masimo); and cerebral oxygen saturation (rSo_2_) by near-infrared spectroscopy (NIRS), also from a forehead sensor (O3 Regional Oximetry, Masimo). Additionally, respiratory rate (RR), inspired fraction of o_2_ (Fio_2_) and co_2_ (Fico_2_), etco_2_, expired ventilation per minute (V̇e), and tidal volume (Vt) were sampled continuously and acquired together via the portable telemetric gas analysis system. NIRS sensors were placed at a standardized frontotemporal location, high on the forehead to avoid any influences from the frontal or sagittal sinuses. The forehead Spo_2_ sensor was applied on the opposite side from the NIRS.

Before and after burial, participants were asked to rate a set of symptoms, including anxiety and stress, cold, discomfort, drowsiness, dyspnea, hallucinations, headache, nausea, and tachycardia, using a visual analogue scale (VAS).^9^ Participants placed a mark on a 100-mm VAS horizontally positioned with the extremes from not at all to all. Participants completed a questionnaire about sensation of chest compression, dyspnea, voluntary change in ventilatory pattern, as well as possible reasons for a request of trial termination (including anxiety, cold, discomfort, dyspnea, tachycardia, and other reasons).

The co_2_ concentration at the end of the exhalation tube was recorded continuously using a portable o_2_ and co_2_ analyzator (Oxycarb-6, Isolcell Industry). Snow density was determined by weighing a predefined snow volume taken with a density cutter both from the area of the air pocket and from the sidewall of the cavity nearby the head position.^2,10^ We calculated the mean cutter density of the 3 samples.

### Statistical Analysis

We used R statistical software version 4.1.1 (R Project for Statistical Computing) to build the database inserting the data of the individual output files of the 4 devices, and we used the TTR library to smooth values of RR, V̇e, Vt, and etco_2_ by means of a moving mean of 30-second intervals (eFigure in Supplement 1). We used SPSS statistical software version 27 (IBM) for statistical analysis. We analyzed the times to reach Spo_2_ less than 84%, as determined by the forehead sensor, and compared intervention and control trials by means of Kaplan-Meier survival curves and a rank test for matched survival data.^11^ To calculate the rank test, we used a modified version of the plr() function of the plac library in R (eAppendix in Supplement 1). We assessed normal distribution of the data with Shapiro-Wilk test and normal Q-Q plots. To compare intervention and control trials, we performed paired *t* tests for normally distributed data and Wilcoxon signed-rank tests for nonnormally distributed data. We used the Friedman test to compare VAS values reported both before and after trials. We used the Benjamini-Hochberg method to adjust *P* values for multiple testing for a false discovery rate of .05. We considered 2-sided *P* < .05 statistically significant. We reported data as mean and SD if they were normally distributed and as median (range) if they were nonnormally distributed. Analyses were conducted between November 2016 and November 2022.

## Results

Two volunteers were unable to participate in either the intervention or control trials, one due to anxiety and the other due to an acute illness (Figure 2). Therefore, a total of 13 individuals (9 men; mean [SD] age, 33 [8] years) participated in both the intervention and control trials and were included in the data analysis (except for 1 control trial, which was excluded due to a suspected air leakage). Participants’ mean (SD) weight was 66 (9) kg and mean (SD) height was 173 (7) cm.

Characteristics of participants and snow are reported in eTable 1 in Supplement 1. Mean (SD) snow density was 384 (42) kg/m^3^. There were no significant differences between intervention and control trials. Of 13 intervention trials, 2 were terminated because of hypoxemia and 11 were terminated by participant request (5 trials for dyspnea; 3 trials for discomfort; 2 trials for cold; 1 trial for an unintentional request). Of 12 control trials, 11 were terminated because of hypoxemia and 1 was terminated by participant request (for anxiety).

### Trial Duration

**Figure 3.**
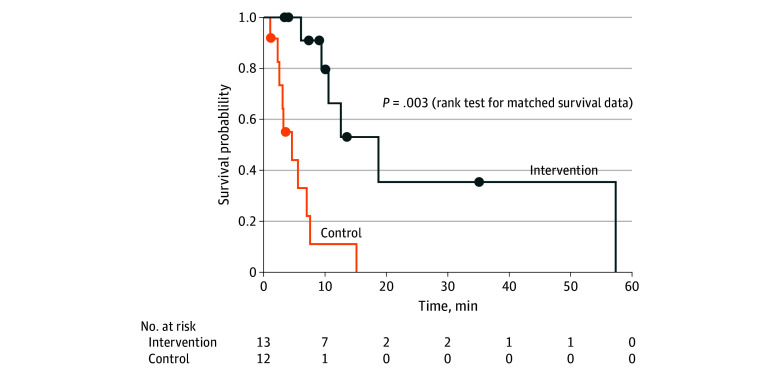
Kaplan-Meier Survival Curves for the Event Peripheral Oxygen Saturation Less Than 84%, Measured by the Forehead Sensor Circles indicate terminations not due to hypoxemia.

**Figure 4.**
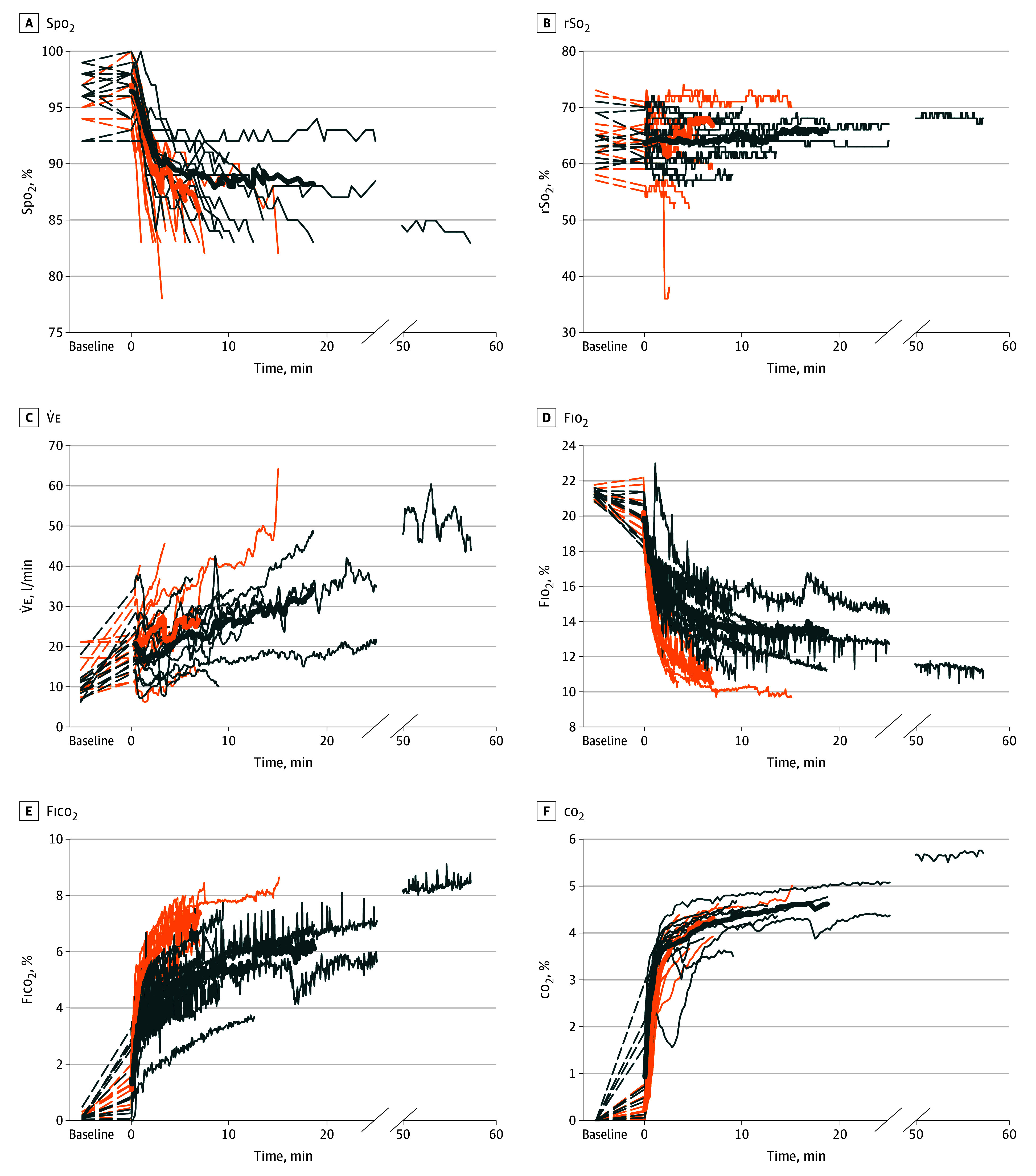
Curves of Individual Physiologic Parameters and Respiratory Gases During the Trials Carbon dioxide (co_2_) concentration was measured at the end of the exhalation tube. Fico_2_, indicates inspired fraction of co_2_; Fio_2_, inspired fraction of oxygen; Spo_2_, peripheral oxygen saturation; rSo_2_, cerebral oxygen saturation; V̇e, expired ventilation per minute. Blue lines represent single intervention trials and orange lines, single control trials. The 2 thicker blue and orange lines represent the mean values of the intervention and control trials, respectively. Mean values are calculated until at least 3 trials are present. Lines are dashed from baseline to start of the trial (0 min).

All participants terminated the trials before 60 minutes. The median (range) burial duration in the intervention trials was 10 (3-57) minutes, compared with 3 (1-15) minutes in the control trials (*P* = .002). Survival curves showed a longer duration of burial in the intervention compared with the control trials for the times to reach Spo_2_ less than 84% (rank test for matched survival data: *P* = .003): among control trials that were ended because of Spo_2_, intervention trials lasted a median (range) of 7 (2-52) minutes longer, corresponding to a median (range) increase of 191% (24%-1130%) (Figure 3). The difference between end of trial and baseline co_2_ concentration at exit of the exhalation tube of the AAPD was higher compared with the concentration in the 0.5-L air pocket of the control trial (mean [SD] difference, 4.4% [0.5%] vs 3.8% [0.8%]; *P* = .04) (Figure 4F; eTable 1 in Supplement 1).

### Physiological Parameters and Respiratory Gas Concentrations

We report physiological parameters and respiratory gas concentrations of each trial in Figure 4 and eTable 1 in Supplement 1. The intervention trials, compared with the control trials, had slower rates of decrease in Fio_2_ (mean [SD] rate, −0.8 [0.4] %/min vs −2.2 [1.2] %/min) and rates of increase in Fico_2_ (mean [SD] rate, 0.5 [0.3] %/min vs 1.4 [0.6] %/min) (Table). The rate of decrease in Spo_2_ was slower in the intervention compared with the control trials (mean [SD], −1.3 [1.0] %/min vs −4.2 [2.5] %/min). The rate of increase in etco_2_ was slower in the intervention compared with the control trials (mean [SD] rate, 0.9 [0.6] mm Hg/min vs 1.8 [0.6] mm Hg/min) (Table). Similarly, the intervention trials, compared with the control trials, had slower rates of increase of Vt (mean [SD] rate, −0.01 [0.04] L/min vs 0.13 [0.14] L/min) and V̇e (mean [SD] rate, 0.5 [1.0] L/min^2^ vs 3.9 [2.6] L/min^2^). There were no statistically significant differences in the rates of change of rSo_2_, HR, or RR (Table).

**Table.** Rates of Change in Respiratory Measures From Start to End of the Trial

Parameter	Trial outcome, mean (SD)		*P* value^a^
	Intervention	Control	
Spo_2_, %/min	−1.3 (1.0)	−4.2 (2.5)	.02
rSo_2_, %/min, median (range)	−0.1 (−0.9 to 1.8)	−0.3 (−8.4 to 0.7)	.20
HR, beats/min^2^	0.2 (2.0)	−0.6 (10.1)	.80
RR, breaths/min^2^	0.5 (0.5)	0.8 (1.0)	.30
Vt, L/min	−0.01 (0.04)	0.13 (0.14)	.02
V̇e, L/min^2^	0.5 (1.0)	3.9 (2.6)	.007
Fio_2_, %/min	−0.8 (0.4)	−2.2 (1.2)	.02
Fico_2_, %/min	0.5 (0.3)	1.4 (0.6)	.02
etco_2_, mm Hg/min	0.9 (0.6)	1.8 (0.6)	.02

Abbreviations: etco_2_, end-tidal carbon dioxide; Fico_2_, inspired fraction co_2_; Fio_2_, inspired fraction of oxygen; HR, heart rate; RR, respiratory rate; Spo_2_, peripheral o_2_ saturation; rSo_2_, cerebral o_2_ saturation; V̇e, expired ventilation per minute; Vt, tidal volume.

^a^ Comparisons between intervention and control trials were analyzed by means of paired samples *t* test, except for rSO_2_ by means of Wilcoxon signed-rank test. *P* values were adjusted for a false discovery rate of .05 using the Benjamini-Hochberg method.

### Questionnaires and VAS Scores

Overall, in all 25 trials, participants reported chest compression in 9 trials, dyspnea in 11 trials, and changes in ventilatory pattern in 17 trials. VAS scores of subjective evaluations of different symptoms are reported in eTable 2 in Supplement 1. Participants reported an increase of anxiety and stress, cold, dyspnea, and discomfort. Symptoms of discomfort were not reportedly elevated in the intervention trials compared with the control trials, despite the longer duration of the intervention trials.

## Discussion

This comparative effectiveness trial found that the new AAPD was associated with delayed development of hypoxemia and hypercapnia in supine participants critically buried in simulated avalanche debris. The AAPD was capable of separating exhaled from inhaled air, slowing down the rates of decrease of Fio_2_ and increase in Fico_2_. AAPD use also slowed the rate of increase of Vt and V̇e, therefore increasing tolerated burial time. These findings suggest that AAPD use may be indicated to improve avalanche burial outcomes by allowing more time for rescue attempts by companions or prehospital emergency medical services. Although most participants reported dyspnea and chest compression, they did not report higher levels of anxiety and stress, cold, discomfort, or dyspnea in the intervention trials, despite the longer duration of burial in these trials.

A 2000 snow-burial study^5^ found that an AAPD diverting expired co_2_ away from a 500-cm^3^ artificial inspiratory air pocket allowed participants to breathe for up to 60 minutes without developing critical levels of hypoxemia (ie, Spo_2_ <84%). Our study confirms these results with a newer, technically advanced AAPD that, instead of using a plastic mesh air pocket, simply separates exhaled from inspired air with two 1-way antifreeze-ball valves, directing the exhaled air into the backpack (dissipating exhaled air into the snowpack behind the user). We have experimentally shown that the AAPD effectively diverted co_2_ into the snowpack, resulting in a slower decrease of Fio_2_ and increase in Fico_2_ in the intervention trials compared with the control.

All participants terminated the trials before 60 minutes, in most cases because of a sensation of dyspnea and chest compression. Such results were unusual compared with other simulated avalanche burial trials and could be related to the supine burial position, which was different than what has been used in previous investigations.^5,6,12^ The supine position might even increase the risk of asphyxiation by restricting chest wall expansion directly owing to the weight of the snow on the chest and indirectly by limiting caudal diaphragm movement. A study by Stalsberg et al^13^ reported that chest compression was a regular concern in avalanche survivors; additionally, hemothorax and marked lung injuries were frequently observed as autopsy findings in persons killed by avalanches. Restricting respiratory motion could also impair the efficacy of devices like an AAPD; therefore, both the position the devices are tested in and the burial depth should be considered in future investigations. A 2016 study^1^ did investigate the association between burial depth and survival and found that mortality rate was independently associated with burial depth, with mortality risk increasing by almost 5 times if an individual was buried more than 120 cm deep, compared with 40 cm or less.^1^

To our knowledge, our study was the first to measure respiratory parameters under simulated avalanche debris in participants buried in a supine position. Compared with baseline levels, participants increased ventilation in the first 30 seconds of burial. During the control trials, V̇e increased nearly 8 times faster than during the intervention trials. This increased ventilatory drive was possibly induced by an increased co_2_ content in the air pocket, stimulating V̇e, favoring quicker o_2_ consumption and contributing to further co_2_ accumulation. To our knowledge, 1 other study^14^ has been performed with supine participants breathing into an artificial air pocket, but with the body out of the snow. Similar to our trial, a study by Wik et al^14^ found that in the control trial, Vt and V̇e increased significantly, with a lower Fio_2_ and a higher Fico_2_. The faster decrease in Fio_2_ might increase risk of asphyxiation.

Our data described for the first time, to our knowledge, cerebral rSo_2_ determined by NIRS in participants under simulated avalanche debris. Although the rate of rSo_2_ decrease was slower in the intervention than in the control group, our data did not show a statistically significant difference between groups. The reason could be that for safety reasons, the trials were terminated while the participants were still only mildly hypoxemic. We previously described that rSo_2_ decreased in participants breathing into an artificial air pocket but only when they desaturated to an Spo_2_ of less than 75%.^15^ Delaying desaturation, AAPD might prevent an insufficient o_2_ supply to the brain.

Although we acknowledge that other avalanche safety technologies, such as airbags, attempt to prevent burial rather than prolong survival once buried,^16^ the adjusted risk of critical burial is still 47% with noninflated airbags (and 20% with inflated airbags).^17^ Our study provides support for the use of an AAPD for persons buried in avalanches in the supine position. The combination of an airbag with an AAPD may further increase avalanche survival rates by allowing companions or prehospital emergency medical services more time to successfully extricate an individual critically buried by an avalanche.^1,18^ In fact, the use of an AAPD is currently recommended by Wilderness Medical Society practice guidelines for prevention and management of avalanche and nonavalanche snow burial accidents^19^ but remains to be clinically investigated.

### Limitations

This study has some limitations. This is an experimental study; therefore, the main limitation remains that the results cannot be simply transferred to a real-world scenario. It is not known whether a recreationist would be able to reach or even quickly and safely insert an AAPD mouthpiece during the course of an avalanche burial. Equally, for safety reasons, participants were only buried at a depth of approximately 50 cm, meaning that we cannot use our data to extrapolate to an avalanche scenario with a higher burial depth that could further restrict ventilation. Furthermore, we were not able to fully respect the randomization list because unpredictable weather changed environmental conditions. This meant that in most trials, the intervention trials preceded the control trials. Despite this, participants did not rate a higher level of anxiety or stress in either trial.

## Conclusions

In this comparative effectiveness trial, we found that this new AAPD was associated with effective delay of the development of hypoxemia and hypercapnia in supine participants buried by simulated avalanche debris. Use of the AAPD may allow a longer burial time before asphyxial cardiac arrest. This might allow longer times for successful rescue by companions or by prehospital emergency medical services. Epidemiological data of winter recreationists equipped with the AAPD should be systematically recorded to estimate their association with avalanche survival.

## Notes

Supplement 1. **eFigure.** Example of Smoothing Applied to Respiratory Rate to Reduce Variability **eAppendix.** Function to Calculate the Rank Test for Matched Survival Data in R **eTable 1.** Trial Duration, Snow Density, Physiological and Ventilatory Parameters, and Respiratory-Gas Concentrations Per Participant and Trial **eTable 2.** Visual Analogue Scale Scores of Symptoms Before and After Trials

Supplement 2.
Data Sharing Statement
