# Supplementary material for: Comparative Effectiveness of an Artificial Air Pocket Device to Delay Asphyxiation in Supine Individuals Critically Buried in Avalanche Debris
Source: JAMA Netw Open. 2023 May 15;6(5):e2313376. doi: 10.1001/jamanetworkopen.2023.13376 (PMC12578492; doi:10.1001/jamanetworkopen.2023.13376)
Supplement: Supplement 1. — eFigure. Example of Smoothing Applied to Respiratory Rate to Reduce Variability eAppendix. Function to Calculate the Rank Test for Matched Survival Data in R eTable 1. Trial Duration, Snow Density, Physiological and Ventilatory Parameters, and Respiratory-Gas Concentrations Per Participant and Trial eTable 2. Visual Analogue Scale Scores of Symptoms Before and After Trials [file jamanetwopen-e2313376-s001.pdf]

Supplemental Online Content

Strapazzon G, Rauch S, Malacrida S, et al. Comparative effectiveness of an artificial air pocket device to delay asphyxiation in supine individuals critically buried in avalanche debris. *JAMA Netw Open*. 2023;6(5):e2313376. doi:10.1001/jamanetworkopen.2023.13376

eFigure. Example of Smoothing Applied to Respiratory Rate to Reduce Variability

eAppendix. Function to Calculate the Rank Test for Matched Survival Data in R

eTable 1. Trial Duration, Snow Density, Physiological and Ventilatory Parameters, and Respiratory-Gas Concentrations Per Participant and Trial

eTable 2. Visual Analogue Scale Scores of Symptoms Before and After Trials

This supplemental material has been provided by the authors to give readers additional information about their work.

© 2023 Strapazzon G et al. *JAMA Network Open.*

eFigure. Example of Smoothing Applied to Respiratory Rate to Reduce Variability

Example of smoothing applied to respiratory rate (RR) to reduce variability. Above progress of original data and below after smoothing by means of a moving average of 30 seconds.

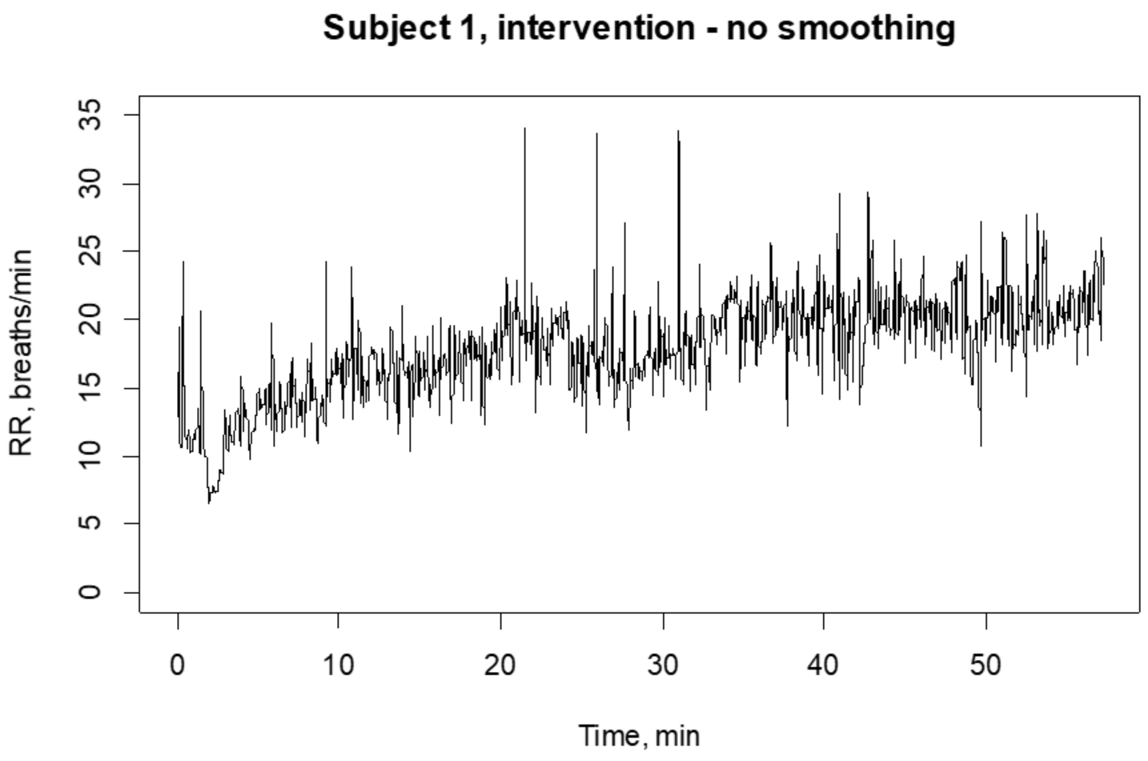

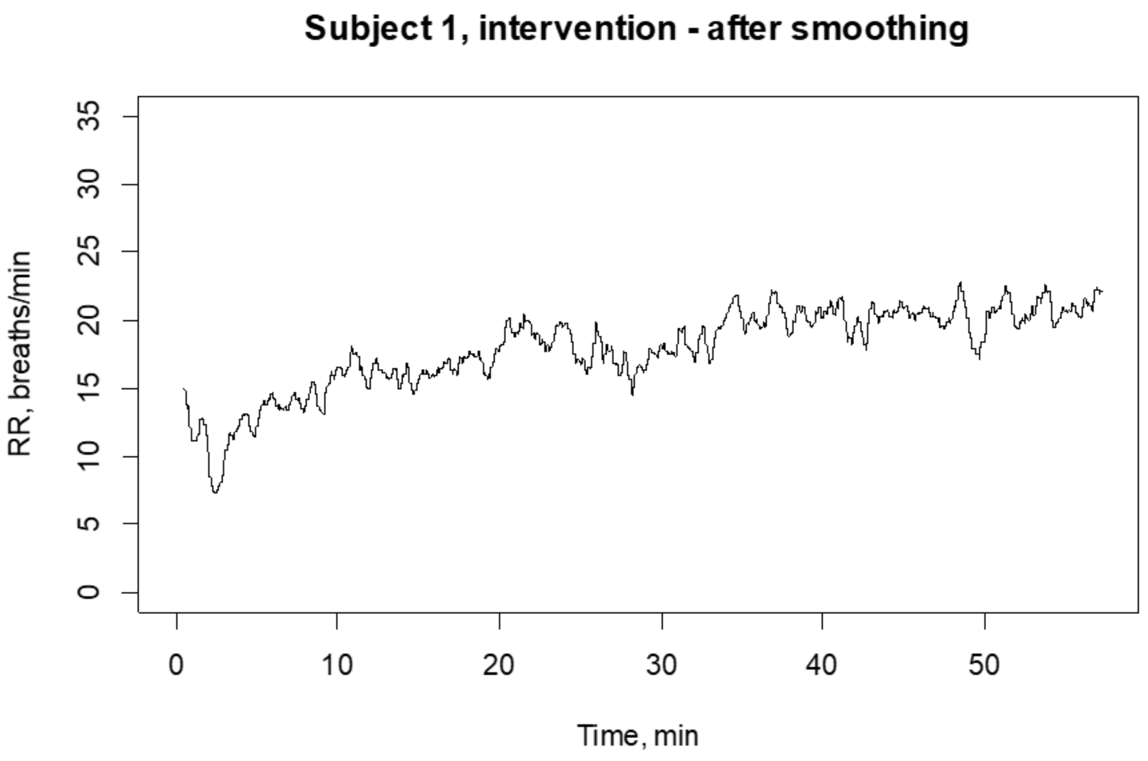

© 2023 Strapazzon G et al. *JAMA Network Open.*

eAppendix. Function to Calculate the Rank Test for Matched Survival Data in R

To calculate the rank test for matched survival data the following function in R was used. The function is a modified version of the function ‘plr()’ of the library plac. The argument `dat` indicates the dataframe, `T1.name` and `T2.name` the names of the two survival time variables and `D1.name` and `D2.name` the names of the two corresponding event indicators.

```
pairedLogRank<-function (dat, T1.name = "T1", D1.name = "D1", T2.name = "T2", D2.name = "D2")
{   n = nrow(dat)
    names(dat) = gsub(T1.name, "T1", names(dat))
    names(dat) = gsub(D1.name, "D1", names(dat))
    names(dat) = gsub(T2.name, "T2", names(dat))
    names(dat) = gsub(D2.name, "D2", names(dat))
    fit.T1 = survival::survfit(Surv(T1, D1) ~ 1, data = dat)
    lambda.T1 = data.frame(et = fit.T1$time, hazard.T1 = fit.T1$n.event/fit.T1$n.risk)
    Y.T1 = stepfun(fit.T1$time, c(fit.T1$n.risk, 0), right = TRUE)
    fit.T2 = survival::survfit(Surv(T2, D2) ~ 1, data = dat)
    lambda.T2 = data.frame(et = fit.T2$time, hazard.T2 = fit.T2$n.event/fit.T2$n.risk)
    Y.T2 = stepfun(fit.T2$time, c(fit.T2$n.risk, 0), right = TRUE)
    et = unique(sort(c(fit.T1$time, fit.T2$time)))
    lambda.diff = merge(lambda.T1, lambda.T2, by = "et", all = TRUE)
    lambda.diff$hazard.T1[is.na(lambda.diff$hazard.T1)] = 0
    lambda.diff$hazard.T2[is.na(lambda.diff$hazard.T2)] = 0
    lambda.diff$h.diff = lambda.diff$hazard.T1 - lambda.diff$hazard.T2
    Hn = Y.T1(et) * Y.T2(et)/(Y.T1(et) + Y.T2(et))/n
    Wn = sqrt(n) * sum(Hn * lambda.diff$h.diff)
    epsilon = rep(0, n)
    X = c(dat$T1, dat$T2)
    delta = c(dat$D1, dat$D2)
    foo = function(x, y) x >= y
    Epsilon = outer(X, X, "foo")
    epsilon = delta[1:n] * colSums(Epsilon[(n + 1):(2 * n), 1:n])/colSums(Epsilon[, 1:n]) - Epsilon[1:n, ] %*% (delta * colSums(Epsilon[(n + 1):(2 * n), ])/(colSums(Epsilon))^2) -
delta[(n + 1):(2 * n)] * colSums(Epsilon[1:n, (n + 1):(2 * n)])/colSums(Epsilon[, (n + 1):(2 * n)]) + Epsilon[(n + 1):(2 * n), ] %*% (delta * colSums(Epsilon[1:n, ])/(colSums(Epsilon))^2)
    epsilon = as.vector(epsilon)
    sigma = sqrt(mean(epsilon^2))
    plr.stat = Wn/sigma
    plr.p = (1 - pnorm(abs(plr.stat))) * 2
    return(list(Stat = plr.stat, P = plr.p))    }
```

© 2023 Strapazzon G et al. *JAMA Network Open.*

eTable 1. Trial Duration, Snow Density, Physiological and Ventilatory Parameters, and Respiratory-Gas Concentrations Per Participant and Trial

Trial	Subject's id	Snow density, kg/m^3	Burial time, min	SpO_2, %			rSO_2, %			HR, beats/min			RR, breaths/min			VT, l			$\dot{V}E$, l/min			FiO_2, %			$FiCO_2$, %			$EtCO_2$, mmHg			CO_2, %		
				BL	Start	End	BL	Start	End	BL	Start	End	BL	Start[1]	End[1]	BL	Start[1]	End[1]	BL	Start[1]	End[1]	BL	Start	End	BL	Start	End	BL	Start[1]	End[1]	BL	Start	End
Intervention	1	360	57.25	92	93	83	64	66	68	100	105	107	15	15	22	0.68	1.45	1.99	10	22	44	21.5	18.5	11.2	0.1	2.8	8.5	34	36	51	0.0	1.6	5.7
	2	430	18.63	99	100	83	61	60	67	97	108	110	19	15	23	0.33	1.89	2.13	6	28	48	21.4	20.0	11.2	0.0	0.9	6.9	31	27	44	0.0	0.5	4.8
	3	400	10.50	96	94	83	71	70	67	106	104	98	12	9	15	0.84	2.02	2.31	10	17	34	21.0	21.4	12.3	0.0	0.1	5.5	25	32	39	0.0	0.7	4.4
	4	340	3.35	99	98	85	59	61	58	86	88	92	13	15	14	0.53	1.08	0.89	7	17	13	20.8	18.5	15.1	0.5	3.3	4.8	37	38	44	NA	NA	NA
	5	420	9.35	98	99	83	63	64	62	77	54	89	10	12	12	1.75	3.02	2.56	18	35	30	21.2	18.2	10.6	0.1	2.9	7.8	22	32	46	0.0	2.1	4.7
	6	350	35.02	98	97	93	65	64	63	104	122	72	16	12	NA	0.58	1.15	NA	9	14	NA	21.2	19.9	NA	0.2	1.9	NA	33	34	NA	0.0	0.1	4.3
	7	350	12.50	96	97	83	60	60	61	106	124	108	10	12	15	1.29	1.75	1.79	12	22	26	21.2	20.7	13.7	0.0	0.0	3.7	16	24	30	0.0	0.7	4.6
	8	340	6.02	96	98	83	63	63	60	81	81	92	17	14	18	0.41	0.99	0.83	7	14	15	21.4	21.4	14.3	0.1	0.4	4.9	32	30	38	0.0	0.1	3.9
	9	340	10.00	96	96	91	69	65	70	112	118	125	15	17	NA	0.61	1.51	NA	9	25	NA	21.6	20.7	NA	0.1	0.8	NA	36	36	NA	0.0	0.1	4.5
	10	NA	9.00	92	92	85	60	61	58	98	93	111	15	14	30	0.58	1.09	0.35	9	16	10	21.1	18.2	14.7	0.1	2.5	4.8	29	32	35	0.0	3.0	3.5
	11	360	3.98	97	98	89	65	61	68	62	83	67	21	14	16	0.57	1.35	1.55	12	19	25	21.0	20.6	15.1	0.1	0.3	4.1	29	33	38	0.0	0.4	4.1
	12	430	7.30	97	94	85	69	70	66	62	70	86	18	17	21	0.63	1.02	1.41	11	17	29	21.4	19.8	12.7	0.1	0.8	6.0	30	36	43	0.0	0.1	4.5
	13	340	13.50	98	98	85	62	63	62	114	137	114	17	14	23	0.65	1.63	1.41	11	24	33	21.3	18.6	12.3	0.2	2.7	6.0	31	34	40	0.0	1.9	4.4
	mean[2], all	372	10.00	96	96	85	64	64	64	93	99	98	15	14	19	0.73	1.54	1.57	10	21	28	21.3	19.7	13.0	0.1	1.5	5.7	30	33	41	0.0	0.9	4.4
	mean[2], no subject 9	375	9.93	97	97	85	64	63	63	91	97	95	15	14	19	0.74	1.54	1.57	10	20	28	21.2	19.6	13.0	0.1	1.5	5.7	29	32	41	0.0	1.0	4.4
Control	1	380	5.52	96	96	83	65	67	64	89	105	94	13	15	17	0.76	1.55	1.98	10	23	34	21.2	20.9	10.4	0.3	0.6	8.0	33	35	44	0.0	0.2	4.5
	2	420	15.05	99	98	82	73	70	70	86	100	109	22	13	27	0.53	1.54	2.40	12	20	64	20.9	19.2	9.7	0.2	1.3	8.7	33	36	45	0.0	0.8	5.0
	3	340	2.22	95	96	83	72	71	62	110	107	108	11	9	NA	1.58	2.01	NA	17	17	NA	21.2	20.0	11.6	0.0	1.1	6.1	28	35	38	0.0	0.3	3.6
	4	NA	1.12	95	97	94	63	64	64	76	88	89	17	20	21	0.58	0.89	1.13	10	18	23	20.9	NA	NA	0.0	NA	NA	35	34	36	0.0	0.1	2.0
	5	440	7.50	95	97	82	62	66	71	92	107	101	13	13	14	1.62	1.74	2.69	21	23	38	20.9	18.8	10.1	0.1	2.0	7.8	21	36	45	0.0	0.2	4.6
	6	440	6.95	97	100	83	62	60	59	83	113	77	14	11	15	0.68	1.00	0.92	9	12	14	21.2	19.7	11.4	0.1	1.6	6.2	33	31	41	0.0	0.5	3.9
	7	NA	1.02	94	93	83	65	62	61	77	65	93	11	13	16	1.26	2.59	2.56	14	33	40	20.9	NA	NA	0.0	NA	NA	25	39	42	0.0	0.0	3.3
	8	NA	2.50	99	98	83	59	59	38	73	104	94	21	19	18	0.36	0.63	1.04	8	12	19	20.9	NA	NA	0.0	NA	NA	27	31	36	0.0	0.0	3.2
	9	NA	-	98	-	-	71	-	-	87	-	-	17	-	-	0.72	-	-	12	-	-	20.9	-	-	0.0	-	-	33	-	-	0.0	-	-
	10	370	3.15	97	100	78	57	55	53	89	125	77	17	14	20	0.55	1.47	1.43	9	20	29	21.5	21.8	10.6	0.2	0.0	6.0	28	28	36	0.0	0.2	3.7
	11	430	3.53	96	98	84	66	64	65	70	92	73	18	17	20	0.54	0.82	2.31	10	14	46	20.8	19.8	10.5	0.3	1.3	7.4	30	35	43	0.0	0.2	4.4
	12	450	3.02	94	94	83	67	66	67	101	87	97	13	20	20	1.62	1.05	1.84	21	21	37	20.9	19.3	10.5	0.1	1.6	6.7	21	35	40	0.0	0.1	4.0
	13	340	4.55	98	97	83	58	56	52	106	132	98	18	21	NA	0.64	1.46	NA	11	30	NA	21.8	22.2	NA	0.1	0.5	NA	25	26	NA	0.0	0.2	3.7
	mean[2], all	401	3.34	96	97	83	65	63	61	88	102	93	16	15	19	0.88	1.40	1.83	13	20	34	21.1	20.2	10.6	0.1	1.1	7.1	29	33	41	0.0	0.2	3.8
	mean[2], no subject 9	401	3.34	96	97	83	64	63	61	88	102	93	16	15	19	0.89	1.40	1.83	13	20	34	21.1	20.2	10.6	0.1	1.1	7.1	28	33	41	0.0	0.2	3.8

BL, baseline; CO_2, carbon dioxide concentration at the end of the exhalation tube; $EtCO_2$, end-tidal CO_2; $FiCO_2$, inspired fraction of CO_2; FiO_2, inspired fraction of O_2; HR, heart rate; NA, not available; RR, respiratory rate; rSO_2, cerebral oxygen saturation; SpO_2, peripheral oxygen saturation; $\dot{V}E$, expired ventilation per minute; VT, tidal volume.

[1] mean of 30 s. [2] median for burial time.

A hyphen is used to denote those values which were excluded from analysis due to suspected air leakage.

© 2023 Strapazzon G et al. *JAMA Network Open.*

eTable 2. Visual Analogue Scale Scores of Symptoms Before and After Trials

Values are reported as median (range). Comparisons of pre- and post-trial visual analogue scale (VAS) scores of intervention and control trials were performed by means of Friedman test and pairwise comparisons by means of Wilcoxon signed-rank test. P-values were adjusted for a false discovery rate of 0.05 using the Benjamini-Hochberg method.

	Intervention trial		Control trial		*P*-values			
**Symptoms[1]**	**Pre-burial**	**During burial**	**Pre-burial**	**During burial**	**Comparison of pre- and during burial phases of intervention and control**	**Intervention: pre-burial *vs.* during burial**	**Control: pre-burial *vs.* during burial**	**During burial: intervention *vs.* control**
Relaxed (0) - stressed out (1)	0.01 (0 - 0.23)	0.5 (0 - 1)	0.02 (0 - 0.9)	0.48 (0 - 1)	0.002	0.01	0.03	0.28
Warm (0) - cold (1)	0.07 (0 - 0.52)	0.28 (0 - 0.76)	0.02 (0 - 0.48)	0.49 (0.02 - 0.99)	0.006	0.02	0.01	0.46
No drowsiness (0) - drowsiness (1)	0.02 (0 - 0.42)	0.01 (0 - 0.3)	0.02 (0 - 0.85)	0.01 (0 - 0.48)	0.74	NA	NA	NA
Comfort (0) - discomfort (1)	0.02 (0 - 0.19)	0.39 (0 - 1)	0.01 (0 - 0.9)	0.31 (0 - 0.98)	0.005	0.02	0.19	0.14
No dyspnea (0) - dyspnea (1)	0.01 (0 - 0.2)	0.75 (0 - 1)	0 (0 - 0.06)	0.24 (0 - 1)	<0.001	0.007	0.02	0.10
No hallucinations (0) - hallucinations (1)	0 (0 - 0.09)	0.01 (0 - 0.08)	0 (0 - 0.03)	0 (0 - 0.06)	0.75	NA	NA	NA
No headache (0) - headache (1)	0.01 (0 - 0.78)	0.03 (0 - 0.88)	0.01 (0 - 0.22)	0.01 (0 - 0.05)	0.82	NA	NA	NA
No nausea (0) -nausea (1)	0.01 (0 - 0.06)	0 (0 - 0.04)	0.01 (0 - 0.9)	0.01 (0 - 0.04)	0.81	NA	NA	NA
No tachycardia (0) - tachycardia (1)	0.02 (0 - 0.2)	0.25 (0 - 0.99)	0 (0 - 0.8)	0.02 (0 - 0.9)	0.08	NA	NA	NA

NA, not applicable.

[1] in brackets value on the VAS

© 2023 Strapazzon G et al. *JAMA Network Open.*
