# Supplementary material for: Comparative Effectiveness of an Artificial Air Pocket Device to Delay Asphyxiation in Supine Individuals Critically Buried in Avalanche Debris
Source: JAMA Netw Open. 2023 May 15;6(5):e2313376. doi: 10.1001/jamanetworkopen.2023.13376 (PMC12578492; doi:10.1001/jamanetworkopen.2023.13376)
Supplement: Supplement 2. — Data Sharing Statement [file jamanetwopen-e2313376-s002.pdf]

Data Sharing Statement

Strapazzon. Comparative Effectiveness of an Artificial Air Pocket Device to Delay Asphyxiation in Supine Individuals Critically Buried in Avalanche Debris. *JAMA Netw Open*. Published May 15, 2023. doi:10.1001/jamanetworkopen.2023.13376

Data
Data available: Yes
Data types: Deidentified participant data
How to access data: The data that support the findings of this study are included in eTable 1 (per single subject) and further data are available from the corresponding author (giacomo.strapazzon@eurac.edu) upon reasonable request.
When available: With publication

Supporting Documents
Document types: None

Additional Information
Who can access the data: The data will be made available to researchers upon reasonable request.
Types of analyses: Upon reasonable request.
Mechanisms of data availability: After approval of a proposal.
